# Fully integrated wearable impedance cytometry platform on flexible circuit board with online smartphone readout

**DOI:** 10.1038/s41378-018-0019-0

**Published:** 2018-07-30

**Authors:** Abbas Furniturewalla, Matthew Chan, Jianye Sui, Karan Ahuja, Mehdi Javanmard

**Affiliations:** Department of Electrical and Computer Engineering, Rutgers, The State University of New Jersey, New Brunswick, USA

## Abstract

We present a wearable microfluidic impedance cytometer implemented on a flexible circuit wristband with on-line smartphone readout for portable biomarker counting and analysis. The platform contains a standard polydimethylsiloxane (PDMS) microfluidic channel integrated on a wristband, and the circuitry on the wristband is composed  of a custom analog lock-in amplification system, a microcontroller with an 8-bit analog-to-digital converter (ADC), and a Bluetooth module wirelessly paired with a smartphone. The lock-in amplification (LIA) system is implemented with a novel architecture which consists of the lock-in amplifier followed by a high-pass filter stage with DC offset subtraction, and a post-subtraction high gain stage enabling detection of particles as small as 2.8  μm using the 8-bit ADC. The Android smartphone application was used to initiate the system and for offline data-plotting and peak counting, and supports online data readout, analysis, and file management. The data is exportable to researchers and medical professionals for in-depth analysis and remote health monitoring. The system, including the microfluidic sensor, microcontroller, and Bluetooth module all fit on the wristband with a footprint of less than 80 cm^2^. We demonstrate the ability of the system to obtain generalized blood cell counts; however the system can be applied to a wide variety of biomarkers by interchanging the standard microfluidic channel with microfluidic channels designed for biomarker isolation.

## Microfluidics: On-the-go blood monitoring

A flexible and wearable impedance cytometer with bluetooth connectivity offers biomarker counting and analysis. Portable health monitoring is envisioned, whereby flexible devices collect, analyze and assess health, and transmit findings to a user interface. However, this places demanding requirements on hardware and software, especially due to often subtle input signals. A team led by Mehdi Javanmard at Rutgers, The State University of New Jersey now report an impedance cytometer on a flexible substrate that can be used for quantifying blood cells and detecting various biomarkers. Blood samples collected by pin-prick are inserted into a microfluidic channel on the wristband, and the data is then wirelessly communicated to an Android application for assessment. Such a device could be used on the field by health professionals for rapid blood monitoring of patients without requiring bulky lab-based equipment.

## Introduction

Increasingly capable smartphones and cheaper off-the-shelf components are constantly pushing what technology can achieve on-the-go^1^. Robust and powerful electronics are driving progress for medical devices, which can be chronically worn or implanted. With current capabilities of digital technology, data sharing, and cloud processing, scientists envision a virtual medical system for providing continuous patient-centered care remotely. Being able to monitor body health is crucial for early detection of illness, which in turn would allow for more accurate diagnosis, more efficient treatment, and lower morbidity or health repercussions^2^.

However, there are tight budget constraints and medical criteria, which biomedical devices must be approved for by the FDA to enter the market when considering wearability or implantability, such as weight and size, biocompatibility, aesthetic factors, and power consumption^3^. Many procedures have been performed in labs for decades with expensive and bulky equipment, which have yet to be translated to wearable health-monitoring technology. Nevertheless, the market for wearable devices has been rapidly growing due to recent achievements in developing miniaturized sensors^4^. For example, due to the development of incredibly robust and miniature accelerometers with microscale processing, devices such as the Fitbit have entered today’s market for monitoring heart rate and user exercise activity^5^. In addition, a variety of flexible electronics are currently being developed by researchers to monitor perspiration for glucose levels and other biomarkers^6–8^. Flexible materials are suitable for wearable devices as they offer superior portability, durability, and robustness^9^.

In the laboratory setting, microfluidic procedures are commonly employed to gather biomedical information for purposes where only a few tens or hundreds of nanoliters of sample are to be analyzed. Macroscale biomedical systems may rely on sensors that lack precision regarding spatial access to or the size of the biological sample, which can be analyzed; however, microfluidic systems can be automated to routinely perform macromolecular separations of the target sample^10^. Lately, miniature microfluidic technologies, or “lab-on-a-chip” microfluidic systems are under investigation, as they have strong potential as wearable biosensors. A skin-like microfluidic device, for example, was recently developed to detect the pH, glucose, and lactate content in sweat through colorimetric sensing^11^. In addition, microfluidics can be applied to create systems which can interface with the body in real time. For instance, a thread-based toolkit of microfluidic sensors has been developed, which can be embedded within body tissue, including a thread-based sensor developed for monitoring the pH of subcutaneous body fluids^12^.

Flow cytometry is a specialized technology where cells, biomarkers, and particles are quanitified. Cell counting is an application of flow cytometry and can provide significant insight into a patient’s health^13^. A well-known example includes a complete blood count (CBC) test which yields information about low or high red blood cell (RBC), white blood cell (WBC), or platelet levels amongst many other specific biomarker counts. However, there are limitations to how often blood counts can be obtained, especially because blood samples must be analyzed each time by a professional using expensive and bulky equipment primarily located in the laboratory setting. There is a need to achieve portable, user-friendly systems to perform automated blood counts so that patient health can be continuously monitored outside of the lab without the need for professional intervention^14^.

Common approaches for counting cells most commonly employ fluorescence or impedance-based measurements. Fluorescence-based cytometers require the labeling of biological cells with antibodies functionalized with fluorophores. Continuous cell counting has been demonstrated in vivo using fluorescence-based flow cytometers^15^. Unfortunately, existing optical instrumentation required to analyze fluorescent particles is overall bulky and expensive, and the labeling procedure is tedious^16^. Impedance cytometry utilizeselectrical measurements^17^ is an alternative technique, which doesn’t require the labeling procedure, and can be used to detect cells^18^, proteins^19^, and nucleic acids^20^. The market offers powerful and versatile coulter counters^21^, such as CytoFLEX (Beckman Coulter, Inc., Brea, CA, USA), however these benchtop instruments are relatively large in size and have yet to be made portable or wearable.

Alternatively, modern coulter counting has been demonstrated using inexpensive circuitry with miniaturized footprints optimized for application-specific tasks, such as blood cell counting^14^. However, these cytometry systems are not designed to be handled by a patient as they rely on expensive external data acquisition hardware and are not packaged into a convenient, user-friendly product. Furthermore, existing prototypes of portable cytometry systems have been implemented on rigid circuit boards, which cannot be worn or implanted to allow continuous and automated blood counting.

Moving away from expensive data acquisition hardware has been a challenge because microfluidic impedance cytometryrequires reading highly sensitive signals on the scale of nanovolts which fall below the noise level of the environment. Lock-in amplification (LIA) is a method which is used to isolate such small signals, by using phase-sensitive detection (PSD). A voltage at an elevated reference frequency is modulated with the impedance response of the system, and the signal response is demodulated by mixing with the original excitation voltage and applying a narrow band-pass filter around the reference frequency^22^.

Even through using LIA, the resulting signal’s baseline may drift over an extended period, reducing the amount of post-gain amplification, which can be applied to the signal, and demanding the use of powerful data acquisition instrumentation with high-resolution (>16 bit) analog-to-digital converters (ADCs). However, in recent work^23^ we developed a novel analog LIA architecture, adding a baseline drift subtraction stage followed by a high-gain amplification stage to allow a low-resolution (10-bit) ADC on a microcontroller unit (MCU) to sample the data (~1 kHz frequency). Although inexpensive MCUs with high-resolution ADC chips are on the market, more bits per sample poses new challenges regarding processing performance and data transmission speeds. Therefore, using a low-resolution ADC was preferable. The resulting system we developed was inexpensive, had a small footprint, and could accurately detect impedance changes as small as 0.01%. We also used a Bluetooth module to transmit data between the microcontroller and the smartphone, allowing the user to initiate data sampling and plot data results on the smartphone. Although we achieved an easy-to-use user interface, our system still consisted of discrete components and not packaged in a user-friendly manner to promote convenient usage outside of the lab.

In this work, we present a portable and fully integrated system including an LIA, a microfluidic polydimethylsiloxane (PDMS) biosensor, a microcontroller, and a Bluetooth module all compacted onto a flexible circuit board in the form of a low-profile wristband with live smartphone readout through an Android application. Blood samples can be obtained via pin-prick and inserted into the inlet of the microfluidic channel for blood cell counting. A medical professional can access the data remotely after being exported from the smartphone, or a machine learning algorithm in the smartphone application could be used to alert the patient about the possibility of illness.

Additionally, different biomarker counts can be obtained by interchanging different microfluidic devices which isolate a specific cell type. For instance, if one wanted to use the platform to count neutrophil (a type of white blood cell) to monitor for neutropenia (low neutrophil count), a high risk case for cancer patients undergoing chemotherapy^24^, the standard microfluidic PDMS chip used in this work can be replaced with a microfluidic device for neutrophil purification^25^.

An immediate benefit of the system being packaged as a wearable wristband is ultra-portability. A wearable cell or particle quantifier could be utilized in a wide variety of biomedical and environmental applications. As an example of important health applications, a catheter could be could be coupled to our system, and complete blood cell counts (CBCs) could be obtained from patients on demand, similar to how temperature, blood pressure, and pulse oximetry measurements can be currently readily obtained. This would especially be useful in an acute setting for patients undergoing surgery or trauma care, where medical professionals need to make quick decisions based on CBC results. Currently, large amounts of blood must be collected from patients and sent to a lab for analysis for CBC counts. Instead, health workers in hospitals or on the field could wear a blood analyzer on their wrist and move from patient to patient, performing rapid analysis. Ultimately, a wearable platform for continuous personal health monitoring applications could also be envisioned if the pin-pricking mechanism is replaced with minimally invasive microneedle or catheter-based impedance sensors, which continuously sample venous blood without the necessity for long intravenous tubes driven by bulky flow pumps.

In the context of environmental monitoring, a wearable impedance cytometer on the wristband could be utilized by inspectors and workers on the field, where different environments must be sampled for different particle counts, such as inorganic elements in mines, or bacteria and other contaminates in water samples. In difficult conditions where dexterity is reduced (in rivers, cold temperatures, etc.), a wearable device would be especially beneficial for performing quick analysis on-site as opposed to collecting and organizing samples and returning to the lab for analysis.

By modifying the impedance cytometer, so that protein^19^ or nucleic acid^20^ biomarker measurements could be obtained, although beyond the scope of this work, other applications involving continuous monitoring protein biomarkers could be envisioned. For example, following a cardiopulmonary bypass procedure (CBP), it is critical to monitor for biomarkers indicating inflammatory response. A systemic inflammatory response during a CBP can result in “vital organ dysfunction, multi-organ failure, and even death^26^.” Monitoring for complement, neutrophil, and platelet activation help indicate the onset of the systemic inflammatory response, however typical CPB procedures involve sampling periods up to 1 h. In ref.^26^, the authors propose a novel microfluidic immunoassay to collect and sample blood continuously. Adoption of a wearable and fully integrated analyzer could enable continuous biomarker quantification and allow for medical professionals to make decisions based on real-time data. Considering the wide array of medical and environmental applications, where a wearable impedance cytometer could be utilized, efforts were focused on building the core elements of the platform technology, namely the microfluidic biosensor chip, the analog front end and communication circuitry, along with the mobile application for data analysis and display.

We first revisit the system architecture from^23^ in depth, and then we describe the construction of the flexible circuit board. Next, we explain the fabrication of the biosensor, and the mobile user interface for the system. To demonstrate the functionality of the overall platform, we use it to count polystyrene beads, sheep blood cells, and human blood cells.

## Materials and methods

### System overview

**Fig. 1:**
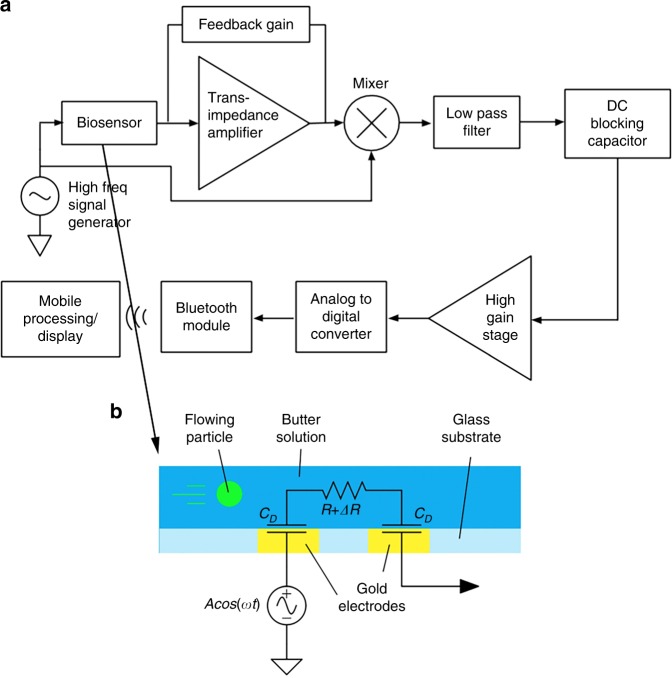
Custom-built analog architecture for impedance cytometry with off-the shelf hardware^23^. **a** System block diagram of cytometer-readout architecture. **b** Lateral view of microfluidic channel, where R represents channel resistance, ΔR is variable resistance from particle flow, and C_D_ is double-layer capacitance

The system diagram is displayed in Fig. 1. We use our custom-built analog architecture^23^, designed to detect highly sensitive impedance changes in a microfluidic channel with low-end hardware.

To perform traditional LIA, a voltage at a high reference frequency is modulated with the microfluidic channel impedance, generating a current signal. The biosensor used in this work relies on an electric field generated between two electrodes within a microfluidic channel, with the baseline impedance representing phosphate buffered solution (PBS), and variable impedance resulting from particle flow through the electric field. A trans-impedance amplifier then amplifies the input current signal and outputs a voltage signal, which is then mixed with the original reference voltage. Finally, a low-pass filter isolates the low-frequency component of the product, which is a low-noise signal proportional to the channel impedance amplitude at the reference frequency^22^. As our channel impedance also varies with time, we designed the low-pass filter cutoff frequency to be larger than the inverse of the transit time of the microfluidic particle, or the time it takes for the particle to transverse the field between electrodes.

After performing traditional LIA on our biosensor, there remains a DC offset within the filtered signal which is in addition to our time-varying signal of interest. The DC offset limits the gain that can be applied to the signal before clipping occurs, and in^23^, we describe the novel use of a DC-blocking stage to subtract the offset and apply a post-subtraction high-gain amplification stage. The result is a highly sensitive architecture, which can be implemented with a small footprint and off-the-shelf components. For an in-depth analysis on the architecture, including the noise analysis and simulation, we refer to the original work^23^. An important note is that the DC-blocking stage causes the positive voltage peak to be followed by a negative voltage peak with the same integrated energy, giving the novel architecture a uniquely shaped peak signature.

Because the analog signal has been amplified over several orders of magnitude, a low-end ADC in a microcontroller chip can sample the data. The microcontroller interfaces with a Bluetooth module paired with a custom developed smartphone application. The application is used to initiate data sampling, and for data processing, readout and analysis.

### Flexible circuit description

**Fig. 2:**
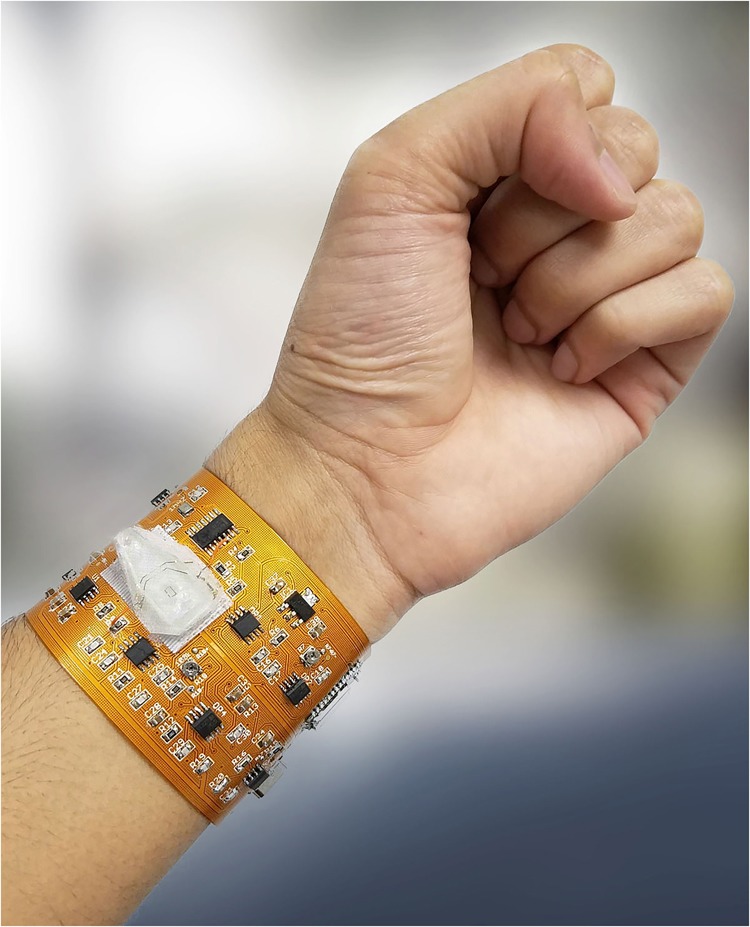
Wearable cytometry system on flexible PCB with integrated microfluidic PDMS chip, microcontroller, and BLE readout to smartphone (not shown)

We have implemented the architecture as a seamless and wearable microfluidic platform by designing a flexible circuit on a polyimide substrate in the form of a wristband (manufactured by FlexPCB, Santa Ana, CA, USA) as shown in Fig. 2. All components, such as the batteries, microcontroller, Bluetooth module, and biochip are unified onto one board. The flexible circuit is a two-layer polyimide board with copper traces totaling an area of 8 in². Surface-mount-packaged components were selected to compact the overall footprint and reduce noise.

Lightweight coin cell lithium ion polymer (LIPO) batteries and regulator chips (LT1763 and LT1964 from Linear Technology) were used to provide ±5 V rails. A 1 MHz AC crystal oscillator (SG-210 from EPSON), D flip-flop (74LS74D from Texas Instruments) for frequency division, and passive LC tank was used to generate the 500-kHz sine wave 2 Volt Peak-to-Peak (V_p-p_) signal, which is excited through the biosensor. The glass wafer acting as the substrate for the biosensor was cut around the PDMS slab with a diamond scribe to minimize the dimensions and was attached to the board via micro-hook-tape and micro-loop-tape strips. The electrodes of the sensor interfaced with the board via jumping wires which were first soldered to the circuit’s terminals and then bonded to the sensor’s terminals with conductive epoxy. Removal of the PDMS sensor involves de-soldering the jumping wires from the circuit board, separation of the micro-hook strip adhered to PDMS sensor from the underlying micro-loop strip adhered to the board, and vice versa for the addition of another sensor. A DC-blocking capacitor was added prior to the biosensor to prevent low-frequency power surges from damaging the biosensor while the circuit was being switched on or off. The trans-impedance stage following the biosensor was implemented with a low-noise operational amplifier (TL071CP from Texas Instruments) and a potentiometer in the feedback path for adjustable gain from 0.04 to 0.44. Mixing was achieved with a multiplier (AD835 from Analog Devices). To isolate the component of interest from the product of the mixing stage, a third order Butterworth low-pass filter with a 100 Hz cutoff frequency and 60 dB roll off per decade was designed with another TL071CP op-amp^23^. A DC-blocking capacitor was used for the DC-blocking stage. The last stage of the analog design, the high gain stage, was achieved with two more TL071CP amplifiers. The first stage has a gain of 1000, and the second stage uses a potentiometer to adjust the gain between 100 and 1100. The high gain stage was minimized during the experiment for a net gain of 10^5^.

An ATtiny 85 8-bit microcontroller from Atmel driven by an external 16 MHz on-board crystal was used to sample data. The microcontroller was programmed through the Arduino IDE (ARDUINO.CC) before being assembled on board. The HM-10 Bluetooth Low Energy (BLE) module was used for data transmission to the smartphone, with the module and the breakout circuit integrated on-board.

### Biosensor fabrication

**Fig. 3:**
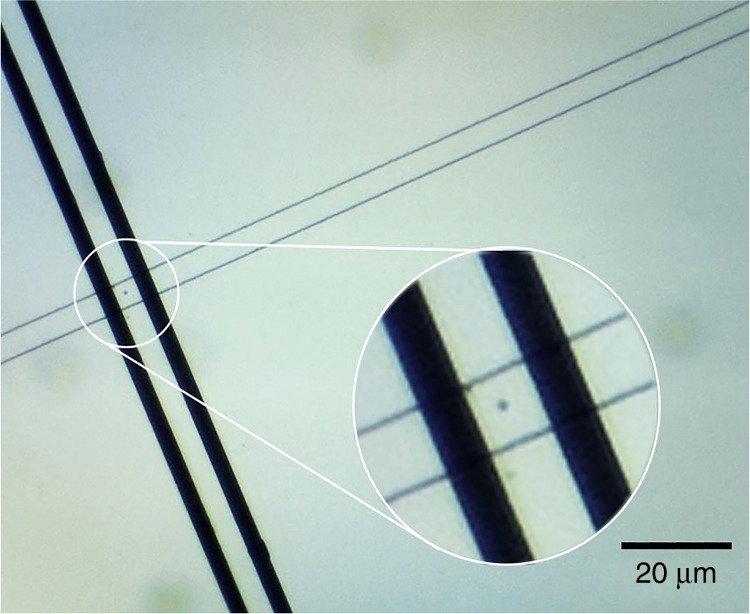
Top view of 30 μm microfluidic PDMS channel pore with sheep blood cells shown flowing across electrodes

The process used to microfabricate our PDMS microfluidic channel for impedance cytometry is a standard one and has been previously reported^27^. We utilized channels with widths of 30 and 50 μm for our experiments, both 10-μm high and 1-cm long. The aerial view of the 30 μm channel is shown in Fig. 3.

The electrode thicknesses we selected were 500 nm Cr followed by 100 nm Au. For the 50 μm channel, the electrode finger design consisted of 10-μm-wide fingers separated by 15 μm. For the 30 μm channel, the width of the fingers was 20 μm separated by 30 μm. The PDMS is intrinsically hydrophobic preventing sufficient flow within the micro-channel^28^. Poly(ethylene glycol)-based polymer containing dihydroxyphenylalanine and lysine (PEG-DOPA-K) was used to improve hydrophilicity and lubricity of the PDMS, improving particle flow^29^.

### Mobile interface

**Fig. 4:**
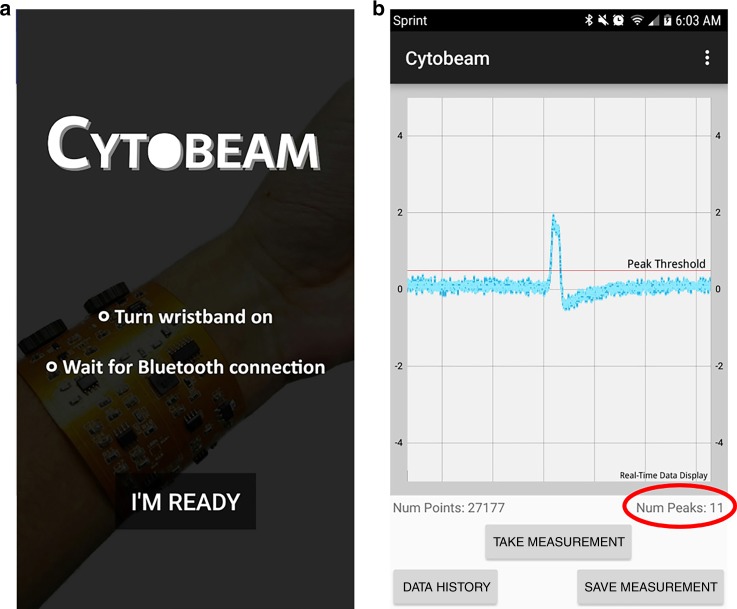
Screenshots from our custom Android application of **a** splash screen **b** live data plot and peak count with buttons to initiate sampling, save the measurement, and plot history data

We developed a Bluetooth Low Energy (BLE)-based application developed for our platform designed to initiate data sampling from the analog circuit, save the data to storage, and plot the data post-sampling. In this work, we update the application to feature online data visualization and peak counting, as well as basic file management capabilities such as history data-plotting and data exporting. Screenshots of the application are depicted in Fig. 4. The application update allows for the smartphone to serve as a replacement of the desktop software for the impedance spectrometer, optimized for the purposes of microfluidic particle counting. A specific advantage of online data readout, which we take advantage of during experimentation includes the ability to simultaneously record optical microscopic results on a computer screen and the electronic data results on a smartphone screen with a 3^rd^ party video recording device, making post-experimental analysis and data alignment more efficient.

### Experimental setup

We used a minimalistic approach to circumvent traditional procedures requiring bulky and expensive equipment with newer procedures that can be performed outside of the lab. As mentioned before, the microfluidic PDMS channel was made hydrophilic by injecting a polyethylene glycol (PEG) solution using a micropipette into the well of the channel, as opposed to traditional oxygen plasma treatment. The use of PEG results in permanently keeping the channel hydrophillic, whereas when treated with oxygen plasma, the PDMS typically returns to its native hydrophobic state within 10s of minutes. Because the device was sufficiently hydrophilic, no external pump was required to generate a steady particle flow. If isolation of a specific blood cell type was desired, instead of using centrifugation (the standard lab approach), passive microfluidic geometries for blood cell sorting have been widely investigated^30^, and can be integrated into microfluidic platforms as needed.

We tested our system with blank PBS, PBS with 3 μm polystyrene beads, sheep blood cells, and human blood cells (<10% WBCs and platelets, >90% RBCs). All samples were diluted in 10 mM PBS with a dilution factor of 20 to reduce to the likelihood of clogging in our simple microfluidic channel, and the channels were filled with 10 mM, pH 7.4 PBS. A channel width of 50 μm was used when counting polystyrene beads and sheep blood cells, and a channel width of 30 μm was used when counting human blood cells obtained from finger pricks, corresponding to channel resistances of 12.5 kΩ and 20.8 kΩ, respectively.

To verify accuracy, an optical compound microscope was used alongside the digital readout system to verify the accuracy of the digitally reported particle counts. For the purposes of optical recording, the biosensor was removed from the surface of the board (while still connected to the system via the jumper cables) and was positioned under the microscope so that the sensor’s electrodes were visible under the field of view as in Fig. 3. A digital camera was mounted onto the microscope lens so that the channel flow could be monitored on the desktop screen.

Simultaneously, the lock-in amplification system was turned on through a power switch. The Bluetooth module was paired with an Android smartphone running our custom Android application. Through the application, the microcontroller was prompted to begin sampling voltage data. The voltage data would then be plotted in live time on the smartphone application.

**Fig. 5:**
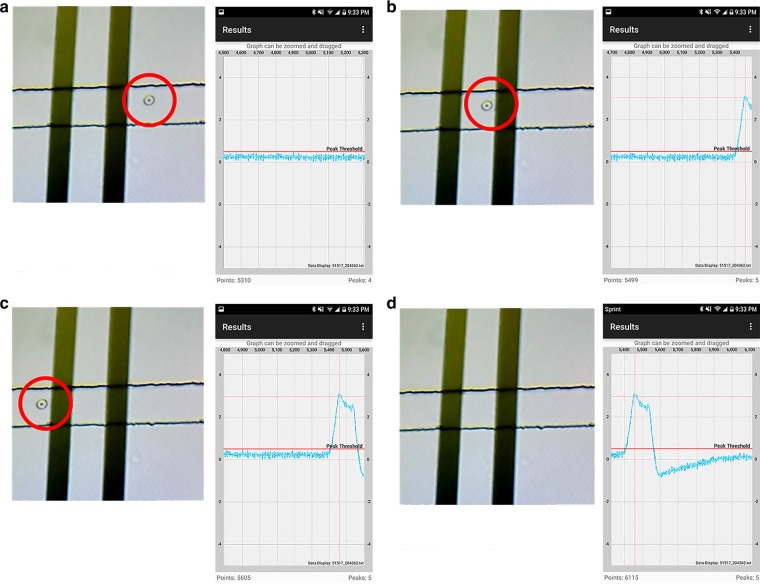
Screenshots from video and cellphone recorded during experiment, combining the optical microscopic view of the sensor and the digital data plotted on the smartphone as human RBC flows past the electrodes from **a**–**d**

As the data was being sampled, a third-party device was used to video record the microscopic view on the desktop screen and the voltage signal on the smartphone, as shown in Fig. 5.

## Results and discussion

**Fig. 6:**
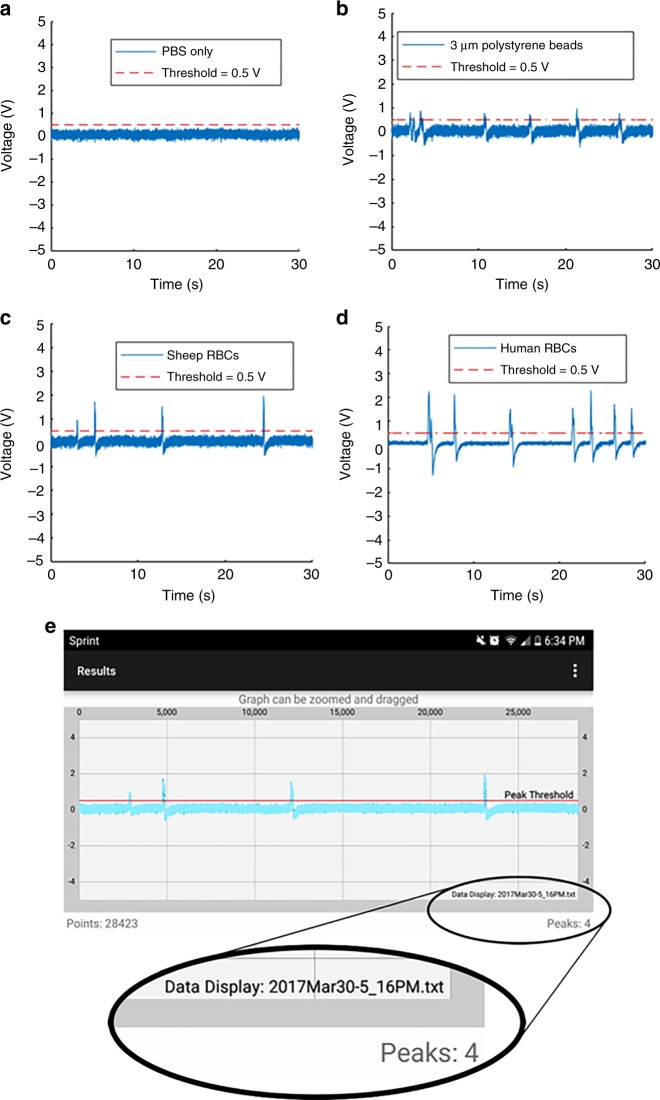
Data exported from smartphone application, measured through wearable LIA with PBS only **a** and 3 μm polystyrene beads **b** and sheep blood cells flowing through 50 μm PDMS channel **c** and human blood cells flowing through 30 μm channel for 30 s **d**. **e** Results from figure **c** data shown on smartphone application

The results of 30 s experiments using blank PBS and PBS with 3 μm polystyrene beads, sheep RBCs, and human blood cells are displayed in Fig. 6. Due to processing limitations of smartphone hardware, an efficient algorithm was targeted to count the number of particles flowing through the channel in live time. Therefore, a straightforward positive voltage threshold-based algorithm of 0.5 V was used to count the number of peaks in the smartphone. To remove false counts created by noise, we set a minimum quota in which four consecutive samples of data were required to be above the threshold to iterate the peak count.

**Fig. 7:**
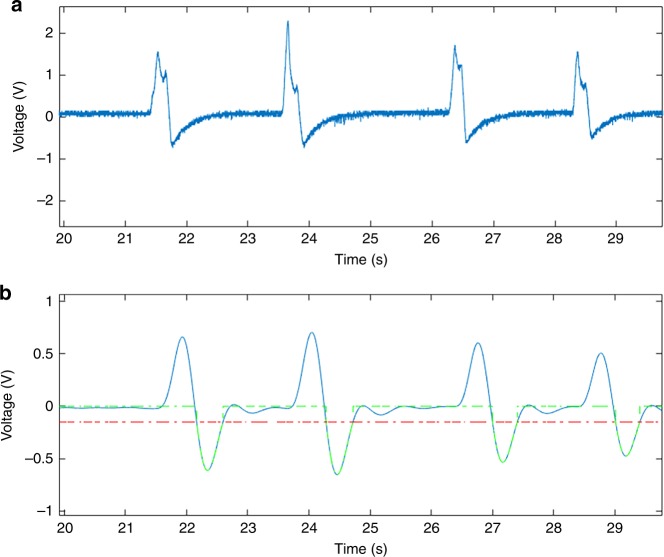
Selected data plotted to MATLAB from 10-minute experiment with human blood cells flowing through a 30-μm-wide channel during an experiment with a duration of 10 min **a** without modification **b** after applying a Butterworth band-pass filter using the Signal Processing Toolbox. The red dashed line represents the negative threshold voltage of −0.2 V, and the green dashed line appears for peaks which included in the final count

For longer experimental samples, with large amounts of data, we performed post-experimental analysis in MATLAB R2016a (MathWorks Inc.) for peak counting. We ran an experiment with a duration of 10 min using human RBCs obtained via pin-prick and diluted in PBS in a 30-μm-wide channel. After the experiment, we exported the data file to a desktop computer. We envision that a patient would similarly export their data remotely to a physician for detailed analysis. The data file was opened in MATLAB and a Butterworth band-pass filter was applied using the Filter Designer from the MATLAB Signal Processing Toolbox. It was necessary to filter out the DC component of the signal to remove drift, and to filter out high frequencies to create a smooth signal, without significantly affecting signal amplitude. The resulting signal is shown in Fig. 7. In the future, the application is envisioned to apply the low-pass filter to the signal in live time.

**Fig. 8:**
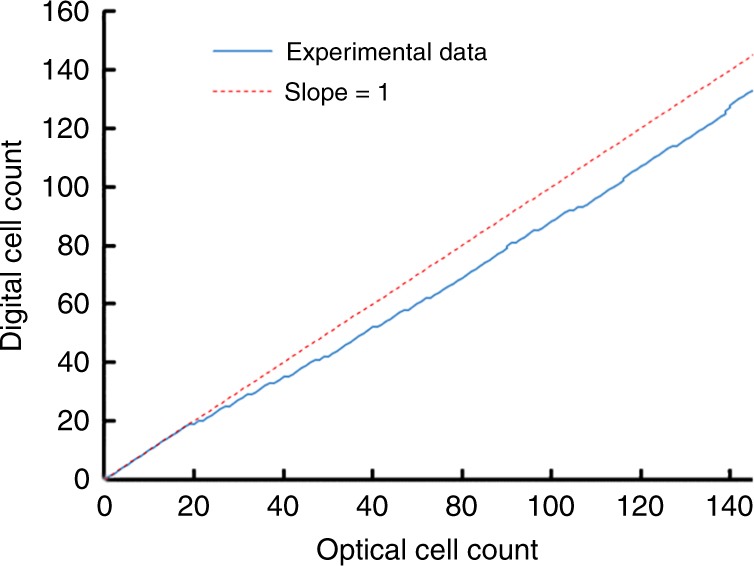
Total optical microscopic count vs. digital system count from 10-minute experiment with human blood cells

The negative voltage overshoot caused by the DC-blocking capacitor helped identify peaks with a higher accuracy than using the original positive voltage peak in the case that cells flow through the electrodes in proximity to each other. Therefore, a negative threshold voltage was applied to count the peaks in MATLAB. The video recording of the experiment was compared to the MATLAB results to analyze optical count vs. digital count. Representative is displayed in Fig. 8.

Threshold-based automated counting only presented difficulty in identifying a peak when multiple cells flowed between the electrodes at a given time resulting in the overlap of separate peaks. Each occurrence of overlapping caused the digital to optical count ratio to drop. However, due to the unique peak signature from our circuit response due to the DC-blocking capacitor, we expect that multiple peak-fitting algorithms such as those used in XPS analysis^31^ can be implemented to obtain more accurate counts.

## Conclusion

We have developed a wearable microfluidic impedance cytometer on a flexible substrate containing a microfluidic biosensor, analog readout hardware, an analog-to-digital data MCU, BLE transmission, and smartphone data processing. Our platform can count the number of blood cells from a pin-prick blood sample pipetted into the standard microfluidic PDMS chip. However, different types of biomarkers can be counted by replacing the standard PDMS chip with specialized microfluidic chips that isolate a specific biomarker. Interchanging the biosensors on the current version of the platform involves de-soldering and re-soldering jumping cables from the biosensor pads to the board, however this can be ultimately replaced with a more user-friendly plug and play packaging interface. The resulting voltage data can be exported and shared with a medical professional for in-depth analysis and can provide vital information to doctors without significantly disrupting a patient’s daily schedule.

In future work, we aim to evaluate the robustness of our platform by sampling data as it is being worn during activity, and we will adjust the circuit architecture, biosensor design, and overall packaging to reduce the effects of motion and environmental disturbance. In addition, we would like to demonstrate the versatility of the system by testing across a range of biosensors and biomarkers. Furthermore, studies will be dedicated to incorporating multi-frequency impedance cytometry and data-driven approaches to discriminate between different cell types. Lastly, our current system requires the user to place samples into the microfluidic channel obtained from a pin-prick, which must be performed at intervals and as opposed to continuous and automated blood counting. Therefore, we envision fabricating minimally invasive microneedle or catheter-based impedance sensors continuously sampling venous blood using a wearable cytometry platform for readout. Bio-systems continuously monitoring human health is the key to early disease prediction and could revolutionize how medical professionals provide treatment to their patients.
